# Volatile compounds released by disturbed and undisturbed adults of *Anchomenus dorsalis* (Coleoptera, Carabidae, Platynini) and structure of the pygidial gland

**DOI:** 10.3897/zookeys.81.1122

**Published:** 2011-02-18

**Authors:** Teresa Bonacci, Pietro Brandmayr, Tullia Zetto, Ida Daniela Perrotta, Salvatore Guarino, Ezio Peri, Stefano Colazza

**Affiliations:** 1 Department of Ecology, Università degli Studi della Calabria – via P. Bucci s.n., 87036, Rende (CS), Ital; 2 Department of Demetra Ed. 5, Università degli Studi di Palermo - Viale delle Scienze, 90128, Italy

**Keywords:** undecane, GC/MS, chemical defences, gland morphology, predation avoidance mechanisms

## Abstract

Volatile compounds produced by adults of Anchomenus dorsalis under undisturbed and disturbed conditions were investigated with an all-glass aeration apparatus. GC-MS analysis of the crude extracts from undisturbed and disturbed adults highlighted four major volatile compounds, undecane, heneicosane, *Z*-9 tricosene and tricosane, of which significantly more undecane was released by disturbed adults compared to undisturbed beetles. The pygidial glands of adults of Anchomenus dorsalis were investigated using light and Transmission Electron Microscopy (TEM). Each gland showed dense aggregates of secretory cells organized into visually distinct lobes; a long collecting canal that drains the secretion towards the reservoir, a bean-shaped double lobed muscular reservoir in which secretion is stored and a short duct (efferent duct) through which the secretion is discharged. The function of the pygidial glands and the possible role played by undecane as a defensive allomone and/or chemical signalling molecule are discussed.

## Introduction

Carabid beetles are known to produce a large variety of defensive substances, and their chemical nature has been elucidated in more than 500 species (Schildknecht et al. 1964, 1968; Eisner et al. 1963; Eisner 1970; Eisner et al. 1977; Moore and Wallbank 1968; Moore 1979; Forsyth 1970, 1972; Kanehisa 1996; Scott et al. 1975; Dazzini-Valcurone and Pavan 1980; Will et al. 2000). The defensive compounds are produced by a pair of glands that open at the tip of the abdomen, known as the “pygidial glands” (Eisner 1958, 1970; Eisner and Aneshansley 1982, 1999; Eisner et al. 1992, 2000). The defensive substances are normally sprayed in the form of large droplets, but in the bombardier beetles the spray mechanism involves a two-chamber system by which benzoquinones are forced against attackers by way of an exothermic reaction (Schildknecht et al. 1968; Eisner et al. 2001). Previous chemical analytical studies on the defensive secretions of several Carabidae species revealed that these substances are blends, mainly characterized by polar organic compounds such as acids, phenols, aldehydes and quinones plus minor non-polar compounds such as ketones, esters and hydrocarbons (Dettner 1987). These chemicals are ejected or discharged mainly when a disturbance stimulus occurs, such as the attack of a predator (Schildknecht et al. 1968; Forsyth 1972; Thiele 1977; Rossini et al. 1997; Eisner et al. 2001). In this case, one or both glands can be discharged almost immediately and the secretion aimed towards the disturbance source (Forsyth 1972). To date, defensive compounds produced by these insects have been analyzed using whole body or droplet extracts but the differences in the quality and amounts of volatiles emitted from the undisturbed and disturbed carabid adults have been not yet been thoroughly investigated.

Anchomenus dorsalis (Pontoppidan, 1763) is a gregarious platynine carabid inhabiting muddy soils and fields across Europe. It is often found in association with species of Brachinus (Juliano 1985; Zaballos 1985; Lindroth 1986; Bonacci et al. 2004a, b; Mazzei et al. 2005; Zetto Brandmayr et al. 2006) and, like Brachinus, it has a bright bicoloured (green-blue and red-brown) coat body that contrasts with the background. These species usually aggregate under heavy stones in open lands with sparse vegetation, such as pasturelands, croplands or in humid, sun exposed soils (Bonacci et al. 2004b; Mazzei et al. 2005). Zetto Brandmayr et al. (2006) described a peculiar “rubbing behavior” of Anchomenus dorsalis towards Brachinus sclopeta (Fabricius, 1792) observed in laboratory conditionsand in natural aggregations, where the individuals (conspecifics and no-conpecifics)live in strinct contact and in peaceful coexistence. Thiele (1977) defines the carabid aggregations as positive intraspecific relationships and as “indications of a type of social behavior”, in which the members of the species are mutually beneficial. Aggregation in ground beetles seems to occur in only very few species and specially between conspicuous and chemically protected species (Thiele 1977; Bonacci et al. 2004b; Zetto Brandmayr et al. 2006). Laboratory investigations carried out by Bonacci et al. (2008) showed that Anchomenus dorsalis and Brachinus sclopeta (which in natural habitats live gregariously), use aposematic colours and warning odours versus natural enemies (Bonacci et al. 2004a, 2006). The authors supposed that the combination of visual and olfactory signals, common in many insect groups (especially aposematic coloured insects, Rothschild and Moore 1987; Moore et al. 1990), can produce a multimodal warning display that, acting along many sensory channels (Rowe and Guilford 1999), increases the antipredatory strategies.

When disturbed, the ground beetles Brachinus dorsalis releases a strong odour (perceived even by humans) (Bonacci, personal observation), and quickly retreat (with dispersal movement that produce a great confusion in the observer) into deeper soil crevices (like the *dilution effect*). Under laboratory conditions, Bonacci et al. (2004a, 2006, 2008) demonstrated that Brachinus dorsalis and Brachinus sclopeta are attacked less by predators, such as Ocypus olens (O. Muller, 1764) (Staphylinidae), Crocidura leucodon (Hermann, 1780) (Insectivora, Soricidae) and Podarcis sicula (Rafinesque, 1810) (Reptilia, Lacertidae) than other carabids used as preys (Juliano 1985; Zaballos 1985; Lindroth 1986; Bonacci et al. 2006, 2008; Zetto Brandmayr et al. 2006). Based on these behavioural studies and observations, experiments were conducted to characterize and quantify the volatile compounds produced by Anchomenus dorsalis upon disturbance. The putative organs producing such defensive compounds, the pygidial glands, were also investigated using light and electronic microscopy.

## Materials and methods

### Insects

Adults of Anchomenus dorsalis were collected by hand from different inter-specific aggregations (each composed by 100–130 individuals) of Brachinus sclopeta and Anchomenus dorsalis found under stones or straw bales in Calabria (Crati Valley, province of Cosenza, 39°35'56"N; 16°15'48"E; elevation: 60 m a.s.l.). Following field collections, monospecific groups were placed in separate plastic cages (30 × 22 × 20 cm) with 4 cm of clay soil in a climatic chamber at 22 °C, photoperiod L/D of 18/6, and fed on veal meat and earthworm pieces (Lumbricus terrestris (Linnaeus, 1758)).

### Air collection of adult volatiles

The collection of volatiles from Anchomenus dorsalis adults was conducted using a horizontal all-glass apparatus 1 l in volume. Humidified and charcoal filtered air was drawn through the apparatus at 0.5 l min-1 by a peristaltic pump for 2 h in a conditioned room at temperature of 22 ± 2 °C. The volatiles produced by experimental groups of 20 individuals of Anchomenus dorsalis adults of both sexes were trapped in glass collectors (6 mm ID) loaded with 600 mg of porapak Q, and held in place by glass wool plugs. Two experimental individual groups were considered: disturbed and undisturbed. Adults were considered undisturbed when they were gently transferred into the glass apparatus and disturbed when, before the start of the aeration, the glass chamber containing the adults was vigorously shaken for 10 seconds (Gómez et al. 2005). Preliminary observations showed that during this time the Anchomenus dorsalis adults released the odour. Five replicates were carried out for each groups: “disturbed” and “undisturbed”. We proceeded in order to avoid any pseudo-replication and each individual was tested once. At the end of the aeration period, collectors were eluted with hexane (400 µl) and the extracts stored at -15 °C until used for GC-MS analysis. Blank aerations were also carried out with the empty apparatus using the same procedure.

### Chemical Analyses

GC–MS analyses were performed using a Hewlett-Packard 5890 GC system interfaced with an HP 5973 quadrupole mass spectrometer detector. As a stationary phase an HP5–MS capillary column (5% diphenyl-95% dimethylpolysiloxane 30 m - 0.2 mm, 0.25 μm film thickness, J&W Scientific, USA) was used. Injector and detector temperatures were 250 °C and 270 °C respectively. Helium was used as the carrier gas. The GC oven temperature program was 60 °C for 5 min, than increased by 10 °C/min to 280 °C. Electron impact ionization spectra were obtained at 70 eV, recording mass spectra from 42 to 550 uma. Compound analysis and identification was carried out using a commercial NIST 2005 mass spectra library search and by comparison with standard analytical grade compounds purchased from Sigma-Aldrich (U.S.A.). Quantitative analysis was carried out for 4 compound identified by GC-MS analysis: undecane, heneicosane, *(Z)*-9 - tricosene and tricosane.

For this analysis the elutes were diluted in 1 ml of hexane using a volumetric flask. Six point calibration curves, using analytical standards undecane, heneicosane, *(Z)*-9 - tricosene and tricosane, in the 0.2–100 ng μl-1 range, were used in order to evaluate the chromatographic response. The mean amount ± SE of each of these compounds was calculated dividing the amount of the compound obtained per replicate per the number of individuals used in each replicate.

### Gland anatomy

For anatomical study by optical microscopy, adult beetles were killed at -15 °C and their abdomens were treated with 10% potassium hydroxide for 4 days before examination of the chitinous structures. The glands were mounted on clean glass slides and observed by optical microscopy equipped with Nomarsky interference contrast and photographed with a Coolpix 4500 camera (Nikon).

For light and transmission electron microscopy (TEM), samples were fixed in 3% glutaraldehyde solution in 0.1 M phosphate buffer (pH. 7.4) for 2 h at 4 °C and post fixed with 3% osmium tetroxide for 2 h. The specimens were then washed in phosphate buffer, dehydrated through graded acetone solutions and embedded in Araldite (Fluka, Buchs, Switzerland). Semithin sections (1 μm) were obtained with a Leica Ultracut UCT ultramicrotome by using glass knives, mounted on clean glass slide and stained with 1% toluidine blue. They were then photographed with the Zeiss Axioskop microscope. For transmission electron microscopy, ultrathin sections (600–900 Ǻ) were prepared using a diamond knife and collected on copper grids (G 300 Cu), contrasted using both lead citrate and uranyl acetate and then examined with a “Zeiss EM 900” electron microscope (TEM). Gland structure terminology follows Forsyth (1972) and Eisner et al. (2001).

### Statistical Analysis

The quantitative analysis to determine differences in the amount of undecane, heneicosane, *(Z)*-9 - tricosene and tricosane recovered from Anchomenus dorsalis adults were compared by t-test (Sokal and Rohlf 1995). The statistical analysis was performed using Statistica for Windows 6.0 (Stat Soft Italia 1997).

## Results

### Chemical analysis of adult volatiles

**Figure 1.**
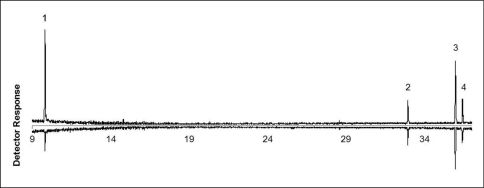
Gas chromatograms of volatile compounds collected from disturbed (up) and undisturbed (down) adults of Anchomenus dorsalis. **1** undecane **2** heneicosane **3** (*Z*)-9 - tricosene **4** tricosane. On the x axis is reported the retention time (minutes). As a stationary phase an HP5–MS capillary column was used. The GC oven temperature program was 60 °C for 5 min, than increased by 10 °C/min to 280 °C.

GC-MS analysis of volatile collections showed that disturbed and undisturbed adults of Anchomenus dorsalis released the same four major volatile compounds: undecane, heneicosane, *(Z)*-9 - tricosene and tricosane (Table 1; Fig. 1). A significant difference between disturbed and undisturbed adults was observed only in the released amount of undecane. An amount of 22.37 ± 8.48 ng (Mean ± SE) were collected from each disturbed adult vs. 0.94 ± 0.29 ng collected from a undisturbed adult (t = 2,52; df = 8; *p* = 0.035). Other chemicals (heneicosane, *(Z)*-9 - tricosene and tricosane) tend to be released more when Anchomenus dorsalis individuals are disturbed but their amount is not statistically significant (Table 1).

**Table 1.** Volatile compounds from Anchomenus dorsalis adults obtained from air collections carried out for 2 hours at 0.5 l min-1. **RT**: retention time at the GC-MS analysis; **df**: degree of freedom.

Compound	R.T. (min.)	Amount (ng) /adult (mean ± SE)		*t*- value	df	p
		Disturbed	Undisturbed			
Undecane	9.82	22.37 ± 8.48	0.94 ± 0.29	2.52	8	0.035
Heneicosane	32.92	1.09 ± 0.96	0.44 ± 0.31	0.63	8	NS
(*Z* )- 9 - Tricosene	35.96	2.19 ± 2.10	0.78 ± 0.70	0.63	8	NS
Tricosane	36.98	0.93 ± 0.86	0.38 ± 0.28	0.60	8	NS

### Gland structure

**Figure 2.**
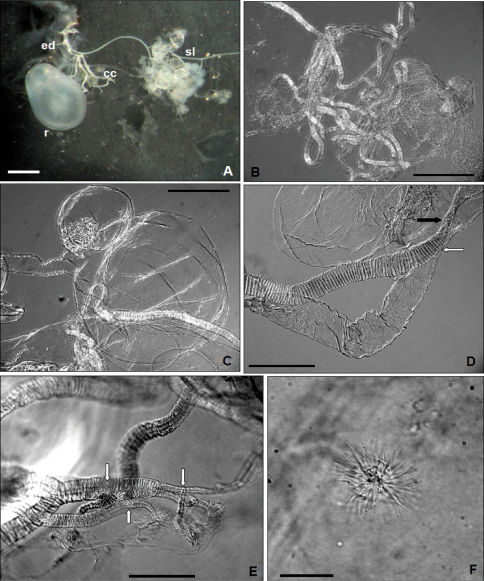
Light microscope: **A** Dorsal aspects of pygidial gland; **ed**, efferent duct; **r**, reservoir; **cc**, collecting canal; **sl**, secretory lobe (Scale bar = 0.5 mm) (not treated with potassium hydroxide) **B** collecting canal (Scale bar = 0.125 mm) **C** reservoir with smooth constriction at about one third from its hind end (Scale bar = 0.125 mm) **D** insertion of collecting canal (black arrow) and efferent duct (white arrow) in the reservoir (Scale bar = 0.05 mm) **E** collecting canal with apical ramifications (white arrows) (Scale bar = 0.05 mm) **F** “floret” (sensu Eisner et al. 2001) (Scale bar = 0.015 mm) (treated with potassium hydroxide).

The pygidial glands of Anchomenus dorsalis are cuticular invaginations of the body wall that open outside immediately behind the eighth abdominal tergite. Each gland consists of a aggregate of secretory cells, an collecting canal, a reservoir and an efferent duct through which the secretion is discharged. The efferent duct open near the abdominal tip to the sides of anus. Each lobe is essentially a ball of cells (fig. 2 A) aligned radially around a central collecting lumen (sensu Forsyth 1970) that carry the secretion towards collecting canal. The overall structure of the secretory lobe and collecting canal resemble a cluster of grapes (fig. 2 A). Each cluster of cells converges to form a long efferent duct that drains the chemical product into a bean-shaped sac (reservoir) in which it is stored (fig. 2 C). The reservoirs extend forward, one along each side of the hindgut. This reservoir or “storage sac” (sensu Rossini et al. 1997) in Anchomenus dorsalis has a smooth constriction at about one third from its hind end, where both the collecting canal as well the efferent duct (sensu Forsyth 1970) converge (fig. 2 C and D). The collecting canal (Forsyth 1970) is a cylindrical tube twice the length of the body of the carabid (fig. 2 B and D), its lumen occupying about one third of the diameter. It carries the secretion from the secretory lobes to the reservoir and shows a continuous and regular spiral ridge along the whole length of its outer surface (fig. 2 D). Each efferent canal (treated with potassium hydroxide) show evident apical ramifications (fig. 2 E) extending towards the center of the secretory lobe. These structures, as observed by Eisner et al. 2001 in Crepidogaster Boheman, 1848 genus and defined by these authors also microtubules (intracellular organelle sensu Rossini et al. 1997), are grouped into convergent clusters, to form tiny individual “floret” that carry the secretion from the secretory lumen (fig. 2 E) to the main collecting canal. The tubules and florets are cuticular and could be isolated readily by potassium hydroxide treatment of the glands.

**Figure 3.**
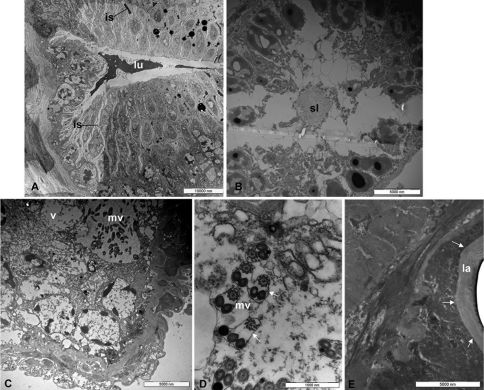
Transmission electron microscope (TEM): **A**, collecting canal with lumen (**lu**); the black arrows show interstitial spaces (**is**) **B** secretory lobe with secretory lumen (**sl**) **C** vesicle (**v**) with microvilli (**mv**) **D** microvilli structure (**mv**) at highest enlargement (the white arrows show the thin lamina) and **E** Inner wall of the reservoir with chitinous basal lamina (**la**) (black arrows) and massive muscle layer around.

Examined by TEM, the wall of the collecting canal is lined by epidermal part (fig. 3 A) that consist of cells connected to each other by micro-canals projecting into the collecting canal lumen. The lumen of collecting canal contain a heterogeneous secretion (fig. 3 A).

Each secretory lobe consist of secretory cells arranged radially around the central lumen. Each secretory cell has an secretory vesicle which is almost as long as the cell itself (fig. 3 B) with a coated membrane and bear many microvilli projecting into the cavity (fig. 3 C). Between the secretory cells are evident (fig. 3 B) the vesicular ducts that carry the secretion in the collecting lumen (fig. 3 B). Each microvillus (fig. 3 D) is formed by three structures: one with a typical spiral shape and other two structures, similar in shape and size. All the structures are enveloped by a thin lamina (fig. 3 D). The inner wall of the reservoir is composed of a thick chitinous layer (basal lamina) (fig. 3 E). A thick muscle layer was found surrounding the reservoir. Likely, the muscles play an important role for the expulsion of the secretory products through the efferent duct. This is composed of muscle bundles that pass spirally around it.

## Discussion and conclusions

The chemical analyses of disturbed and undisturbed Anchomenus dorsalis adults showed that undecane was produced significantly in larger amounts in disturbed individuals. This suggests that this compound (which can be perceived even by humans) could play a prominent role in the chemical defence of the species. The role of undecane as a defensive substance has been widely reported in the Insecta: Acanthomyops claviger (Roger, 1862) (Regnier and Wilson 1968), Formica polyctena Foerster, 1850 (Löfqvist and Bergström 1980), Paratrechina longicornis (Latreille, 1802) (Morgan et al. 2005) and in Oxytelinae rove beetles (Bledius spectabilis Kraatz, 1857, Platystethus arenarius (Fourcroy, 1785), and Oxytelus piceus L.) (Dettner and Schwinger 1982). Although in other carabids, hydrocarbons are generally produced in lesser amounts than polar compounds such as acids, phenols, aldehydes or quinones (Dettner 1987), but the occurrence of undecane in pygidial glands has been described in Pterostichini (Abaris aenea Dejean, 1831, Pseudabarys Chaudoir, 1873, sp), Loxandrini (Loxandrus LeConte, 1852, spp), Morionini (Morion simplex Dejean, 1831, Moriosomus seticollis Straneo, 1985), Catapieseini (Catapiesis Solier, 1835, spp), Perigonini (Diploharpus laevissimus Chaudoir, 1850) and Odacanthini (Colliuris pensylvanica Linnaeus, 1758) (Will et al. 2000). Furthermore, Peschke and Eisner (1987) reported that hydrocarbons produced by carabid species are potentially defensive.

Undecane is an optimal chemical signalling molecule, its molecular weight and polarity combining moderate olfactory efficiency with a sufficiently high vapour pressure to broadcast in the centimetre range when present in microgram quantities or less (Regnier and Wilson 1968). As with a number of carabids (Eisner 1958; Eisner et al. 1963; Will et al. 2000), a defensive spray constituted mainly of undecane appears to be an effective deterrent of predators (Bonacci et al. 2004a, 2008). Moreover, the efficacy of these chemicals is improved in that carabid species that are able to direct their ejections directly against the head or the eyes of the predator (Eisner 1958; Peschke and Eisner 1987).

The laboratory observations of the pygidial glands of Anchomenus dorsalis show that they resemble those of other Carabidae in their structure (Forsyth 1970, 1972), and the form of the reservoir, clearly double lobed, resembles the apparatus of Anthia and Harpalines described by Forsyth (1972). The secretory “florets” show a very small lumen, compared to other tribes of carabid beetles, (Forsyth 1972; Rossini et al. 1997; Eisner et al. 2001), but very little investigations have been done on Platynine (Agoninae) ground beetles. The inner wall of reservoir of pygidial glands in Anchomenus dorsalis shows a thin coat of chitinous tissue which probably preserves the cells by the toxic mixture of chemicals and requires that this secretion must be efficiently isolated from the rest of the body. Another feature of the glands of Anchomenus dorsalis is the extreme length of the collecting canal. This tube seems to be much longer than necessary for the transfer of chemical secretion. Very long collecting canals were found by Forsyth (1970) in Pterostichus madidus (Fabricius) and the author proposed that this feature serves to abridge the back-pressure from the reservoir.

As mentioned above, the defense glands in carabid beetles produce chemical compounds primarily to provide protection against putative predators (Thiele 1977; Will et al. 2000). Nevertheless, in Anchomenus dorsalis, volatile compounds ejected after disturbance could have a double function: repellent function to predators and chemical signalling function for conspecifics. In fact, previous studies showed that the defensive compounds released by a number of disturbed Anchomenus dorsalis individuals is not only a repellent towards natural enemies, but also elicited dispersal behavior in conspecifics (Bonacci et al. 2004a, b, 2006, 2008). Such an intra-specific dispersal function is supported by the gregarious nature of this species; gregariousness and high population density allowing rapid intra-specific communication is generally thought to be necessary for evolution of chemical signalling molecule (Nault and Phelan 1984). It is reasonable to believe that in Anchomenus dorsalis, undecane emission (characterized by strong smell, which can be perceived even by humans) by the pygidial glands causes dispersal movement of individuals inside the aggregation (authors’ personal observations). If the assumption of Blum (1985) is correct (deterrence against predators and intraspecific alarm function of the same compounds being coupled), the chemicals of this carabids evoke an alarm reaction in conspecifics and avoidance behaviour in natural enemies, as showed by Bonacci et al. (2004a, 2006, 2008). Usually Anchomenus dorsalis occurs in dense aggregations of many individuals with other carabids belonging to the genus Brachinus. It can be expected that in species occurring in such masses an adequate defense mechanism has evolved towards potential predators. Brachinus sclopeta producing several defensive chemicals (Zetto Brandmayr et al. 2006) and Anchomenus dorsalis producing a putative chemical signalling molecule, undecane, from the pygidial glands. Assemblages of mixed species that share common predators may experience benefits that are similar to or exceed those of monospecific groups. These benefits may be particularly pronounced if individuals of one species can recognize the alarm signals produced by individuals of other species in the assemblage (in Mathis and Smith 1993).

In summary, undecane and the pygidial glands appear to play a role in the defence mechanism of Anchomenus dorsalis. Further studies will carry on to investigate if undecane emission is able to elicit dispersal and retreating movements both in co-specific and inter-specific groups.

## References

[B1] Bonacci T Aloise G Brandmayr P Zetto Brandmayr T (2004a) Risposte comportamentali di *Crocidura leucodon* (Hermann, 1780) (Insectivora, Soricidae) ai meccanismi antipredatori di alcuni Artropodi. Hystrix, Italian Journal of Mammalogy (Nova Series, Pavia, Italy) 15 (1):73-76

[B2] Bonacci T Mazzei A Zetto Brandmayr T Brandmayr P (2004b) Aposematic aggregtion of carabid beetles (Coleoptera Carabidae): preliminary data. Redia LXXXVII: 243–245

[B3] Bonacci T Massolo A Brandmayr P Zetto Brandmayr T (2006) Predatory behaviour on ground beetles (Coleoptera: Carabidae) by *Ocypus olens* (Müller) (Coleoptera: Staphylinidae) under laboratory conditions. Entomological News 117 (5):545-551

[B4] Bonacci T Aloise G Brandmayr P Zetto Brandmayr T Capula M (2008) Testing the predatory behaviour of *Podarcis sicula* (Reptilia: Lacertidae) towards aposematic and non-aposematic preys. Amphibia-Reptilia 29:449-453

[B5] Blum MS (1985) Alarm pheromones. In: Kerkut GA Gilbert LI (Eds) Comprehensive Insect Physiology, Biochemistry and Pharmacology. Pergamon Press, Oxford, 194–224

[B6] Dazzini Valcurone M Pavan M (1980) Glandole pigidiali e secrezioni difensive dei Carabidae (Insecta Coleoptera). Publicazioni dell’Istituto di Entomologia dell’Università di Pavia, 12:1-36

[B7] Dettner K Schwinger G (1982) Defensive secretions of three oxytelinae rove beetles (Coleoptera: Staphylinidae). Journal of Chemical Ecology, 8 (11):1411-1420 10.1007/BF01403104 24414835

[B8] Dettner K (1987) Chemosystematics and evolution of beetle chemical defense. Annual Review of Entomology 32:17-48

[B9] Eisner T (1958) The protective role of the spray mechanism of the bombardier beetle, *Brachynus ballistarius* Lec. Journal of Insect Physiology 2:215-220

[B10] Eisner T Hurst JJ Meinwald J (1963) Defense Mecanisms of Arthropods. XI. The Structure, Function, and Phenolic Secretions of the Glands of a Chordeumoid Millipede and a Carabid Beetle. Psyche 70:94-116

[B11] Eisner T (1970) Chemical defense against predation in arthropods. In: Sondheimer E Simeone JB (Eds) Chemical Ecology. Academic Press, New York, 235–280

[B12] Eisner T Jones TH Aneshansley DJ Tschinkel WR Silbergleid RE Meinwald J (1977) Chemistry of defensive secretions of Bombardier Beetles (Brachinini, Metriini, Ozaenini, Paussini). Journal of Insect Physiology 23:1383-1386

[B13] Eisner T Aneshansley DJ (1982) Spray aiming in bombardier beetles: jet deflection by the Coanda effect. Science 215:83-85 17790472 10.1126/science.215.4528.83

[B14] Eisner T Attygalle AB Eisner M Aneshansley DJ Meinwald J (1992) Chemical defense of a primitive Australian bombardier beetle: *Mystropomus regularis*. Chemoecology 2:29-34

[B15] Eisner T Aneshansley DJ (1999) Spray aiming in the bombardier beetle: photographic evidence. Proceeding of National Academy of Science, USA 96:9705-9709 10.1073/pnas.96.17.9705 PMC22274 10449758

[B16] Eisner T Aneshansley DJ Eisner M Attygalle AB Alsop DW Meinwald J (2000) Spray mechanism of the most primitive bombardier beetle (*Metrius contractus*). The Journal of Experimental Biology 203:1265-1275 10729276 10.1242/jeb.203.8.1265

[B17] Eisner T Aneshansley DJ Yack J Attygalle AB Eisner M (2001) Spray mechanism of crepidogastrine bombardier beetles (Carabidae; Crepidogastrini). Chemoecology 11:209-219

[B18] Forsyth DJ (1970) The ultrastructure of the pygidial defence glands of the *Pterostichus madidus* F. Journal of Morphology 131: 397–416

[B19] Forsyth DJ (1972) The structure of the pygidial defence glands of Carabidae (Coleoptera). Transactions of the Zoological Society of London 32:249-309

[B20] Gómez J Barrera JF Rojas J Macias-Samano J Liedo JP Cruz-Lopez L Badii MH (2005) Volatile compounds released by disturbed females of *Cephalonomia stephanoderis* (Hymenoptera: Bethylidae): a parasitoid of coffee berry borer *Hypotenemus hampei* (Coleoptera: Scolytidae). Florida Entomologist 88 (2):180-187

[B21] Juliano SA (1985) Habitat associations, resources, and predators of an assemblage of *Brachinus* (Coleoptera: Carabidae) from southeastern Arizona. Canadian Journal of Zoology 63:1683-1691

[B22] Kanehisa K (1996) Secretion of defensive substance by carabidae and Brachinidae. Bulletin Resesearch Institute Bioresources, Okayama Univ. 4: 9–23 [in Japanese]

[B23] Lindroth CH (1986) The Carabidae (Coleoptera) of Fennoscandia and Denmark Fauna. Entomologica Scandinavica, 15, part 2. Scandinavian Science Press, Copenhagen, 223–497

[B24] Löfqvist J Bergström G (1980) Volatile communication substances in Dufour’s gland of virgin females and old queens of the ant *Formica polyctena*. Journal of Chemical Ecology 6 (2):309-320

[B25] Mazzei A Bonacci T Zetto Brandmayr T Brandmayr P (2005) Capacità di aggregazione di Coleotteri geoadefagi, in ambiente ipolitico di suoli argillosi del bioclima mediterraneo arido. Proceedings of XV Congress of the Italian Society of Ecology. Torino, Italy 12–14 September 2005. 1–4

[B26] Mathis A Smith RJF (1993) Intraspecific and cross-superorder responses to chemical alarm signals by brook stickleback. Ecology, 74 (8):2395-2404

[B27] Moore BP (1979) Chemical defense in carabids and its bearing on phylogeny. Carabids beetles: their evolution, natural history, and classification. In Erwin TL Ball GE Whitehead DR Halpern AL (Eds) The Ilague, Junk, 193–203

[B28] Moore BP Wallbank BE (1968) Chemical composition of the defensive secretion in carabid beetles and its importance as a taxonomic character. Proceeding of Royal Entomological Society of London B 37:62-72

[B29] Moore BP Vance Brown W Rothschild M (1990) Methylalkylpyrazines in aposematic insects, their hostplants and mimics. Chemoecology 1:43-51

[B30] Morgan ED Jackson BD Billen J (2005) Chemical secretions of the “crazy ant” *Paratrechina longicornis* (Hymenopetra: Formicidae). Sociobiology 46 (2):299-304

[B31] Nault LR Phelan PL (1984) Alarm pheromones and sociality in pre-social insects. Chemical ecology of Insects. In Bell WJ Cardè RT (Eds) Chapman and Hall, United Kingdom, 237–256

[B32] Peschke K Eisner T (1987) Defensive secretion of the tenebrionid beetle, *Blaps mucronata*: Physical and chemical determinants of effectiveness. Journal of Comparative Physiology A 161:377-388 10.1007/BF00603963 3668879

[B33] Regnier FE Wilson EO (1968) The alarm-defense system of the ant *Acanthomyops claviger*. Journal of Insect Physiology 14:955-970

[B34] Rossini C Attygalle AB González A Smedley SR Eisner M Meinwald J Eisner T (1997) Defensive production of acid formic (80%) by a carabid beetle (*Galerita lecontei*). Proceeding of National Academy of Science, USA 94:6792-6797 10.1073/pnas.94.13.6792 PMC21237 9192644

[B35] Rothschild M Moore B (1987) Pyrazines as alerting signals in toxic plants and insects. In: Laberie V Fabres G Lachaise D (Eds) Insects-Plants. Dordrecht, Holland, 97–107

[B36] Rowe C Guilford T (1999) The evolution of multimodal warning displays. Evolutionary Ecology 13:655-671

[B37] Schildknecht HK Holoubek KH Weiss H Kramer H (1964) Defensive substances of the Arthropods, their isolation and identification. Angewandte Chemie International Edition 3:73-82

[B38] Schildknecht H Maschwitz E Maschwitz U (1968) Die Explosionschemie der Bombardierkäfer (Coleoptera, Carabidae) III. Mitteilung: Isolierung und Charakterisierung der Explosionskatalysatoren. Zeitschrift für Naturforschung. 23b: 1213–1218 4387200

[B39] Scott PD Hepburn HR Crewe RM (1975) Pygidial defensive secretions of some carabid beetles. Insect Biochemistry 5 (6):805-811

[B40] Sokal RR Rohlf FJ (1995) Biometry. 3rd ed. W.H. Freeman, New York, 887 pp.

[B41] Stat Soft Italia (1997) Statistica per Windows. User’s Manual. Statsoft Italia, Vigonza

[B42] Thiele HU (1977) Carabid beetles in their environments. A study on habitat selection by adaptations in physiology and behavior. Springer-Verlag, Berlin, Heidelberg, New York, 369 pp.

[B43] Will KW Attygalle AB Herath K (2000) New defensive chemical data for ground beetles (Coleoptera: Carabidae): interpretations in a phylogenetic framework. Biological Journal of the Linnean Society 71:459-481

[B44] Zaballos P (1985) Paralelismo fenológico en *Brachinus variventris* Schaufuss, 1862 y *Anchomenus dorsalis* (Pontoppidan, 1963). (Coleoptera Carabidae). Actas do II Congresso Ibérico de Entomologia, Lisboa, Ispagna

[B45] Zetto Brandmayr T Bonacci T Massolo A Brandmayr P (2006) What is going on between aposematic carabid beetles? The case of *Anchomenus dorsalis* (Pontoppidan 1763) and *Brachinus sclopeta* (Fabricius 1792) (Coleoptera Carabidae). Ethology Ecology and Evolution 18:335-348

